# A critical appraisal of the ISGLS definition of biliary leakage after liver resection

**DOI:** 10.1007/s00423-022-02746-8

**Published:** 2023-02-03

**Authors:** Svenja Sliwinski, Jan Heil, Josephine Franz, Hanan El Youzouri, Michael Heise, Wolf O. Bechstein, Andreas A. Schnitzbauer

**Affiliations:** 1 Department for General, Visceral and Transplant Surgery, University Hospital Frankfurt, Goethe-University Frankfurt/Main, Theodor-Stern-Kai 7, 60590 Frankfurt/Main, Germany; 2 Institute of Physiology, University of Zurich, Zurich, Switzerland

**Keywords:** Biliary leakage, Bile leakage, ISGLS, Morbidity, Liver surgery

## Abstract

**Purpose:**

The International Study Group of Liver Surgery (ISGLS) defined post-hepatectomy biliary leakage as drain/serum bilirubin ratio > 3 at day 3 or the interventional/surgical revision due to biliary peritonitis. We investigated the definition’s applicability.

**Methods:**

A retrospective evaluation of all liver resections over a 6-year period was performed. ROC analyses were performed for drain/serum bilirubin ratios on days 1, 2, and 3 including grade A to C (analysis I) and grade B and C biliary leakages (analysis II) to test specific cutoff values.

**Results:**

A total of 576 patients were included. One hundred nine (18.9%) postoperative bile leakages occurred (19.6% of the whole population grade A, 16.5% grade B/C). Areas under the curve (AUC) for analysis I were 0.841 (day 1), 0.846 (day 2), and 0.734 (day 3). The highest sensitivity (78% on day 1/77% on day 2) and specificity (78% on day 1/79% on day 2) in analysis I were obtained for a drain/serum bilirubin ratio of 2.0. AUCs for analysis II were similar: 0.788 (day 1), 0.791 (day 2), and 0.650 (day 3). The highest sensitivity (73% on day 1/71% on day 2) and specificity (74% on day 1/76% on day 2) in analysis II were detected for a drain/serum bilirubin ratio of 2.0 on postoperative day 2.

**Conclusion:**

Biliary leakages should be defined if the drain/serum bilirubin ratio is > 2.0 on postoperative day 2.

## Introduction

Biliary leakage is one of the leading causes of postoperative morbidity after liver resection. The International Study Group for Liver Surgery (ISGLS) defined post-hepatectomy biliary leakage as a drain bilirubin to serum bilirubin ratio ≥ 3 at day 3 after resection or later. Additionally, a biliary leakage was present, in case of an interventional/surgical revision due to biliary peritonitis. The incidence of biliary leakage following liver resections varies between 3 [1] and 33% [2] depending on the complexity of the resection. Several studies identified various factors that may lead to biliary leakage, such as compromised blood supply, excessive dissection at the level of the bile duct, preoperative biliary stenting, and small caliber bile duct [3–5].

In the majority of the literature available, the mortality associated with biliary leakages has decreased during the last years and accounts for around or below 3% [1, 6, 7]. However, the outcome can be severe, resulting in biliary peritonitis, biloma, increased health care costs and prolonged hospitalization, and, albeit rarely, death [3, 6, 8, 9]. In general, mortality rates after liver surgery remain high [10]. It is therefore important to recognize a biliary leakage as early as possible to prevent further complications. Thus, a specific definition used internationally should be applied. Our aim was to evaluate the accuracy of the current ISGLS definition of biliary leakage after liver resection due to any indication as a retrospective study of a large study population at a university hospital and to assess potential risk factors.

Since symptoms of biliary leakage are non-specific—which can delay adequate treatment—an early diagnosis is key. We therefore questioned the current definition by the ISGLS. Since this definition is not used as a worldwide standard, there seems to be ambiguity about its reliability, which necessitates further investigation. Several other definitions exist, for example, bile-stained fluid > 50 ml in the abdominal drain more than 24 h after surgery, proven radiologically or at relaparotomy [7], the presence of persisting bile-stained effluent from an abdominal drain or leakage detected on radiological imaging (endoscopic retrograde cholangiography—ERC), the occurrence of a bile collection drained percutaneously [11], or the drainage of bile from the abdominal wound and drain with the level of total bilirubin in the discharge fluid more than 5 mg/ml or three times the serum level [4]. This diverse selection of definitions also prompted us to conduct further research.

## Patients and methods

### Data management and statistical analysis

Data were extracted from the electronic patient charts. The items required are displayed in Tables 1 and 2. In case a patient had a biliary drainage, serum and drain bilirubin values were detected on days 1, 2, and 3. If the serum bilirubin to drain ratio was ≤ 3, the drain was removed. In case it was higher, the drain remained in situ and values were repeated. In case the bile leakage was macroscopically present for more than 4 days, or exceeding 350 ml of pure biliary fluid, the patient underwent endoscopic retrograde cholangiopancreatography (ERCP). Early macroscopically evident bile leakages following liver resections with hepaticojejunostomy were reoperated immediately. In case a patient was lost to follow-up, the date of the last known and documented status was used. ROC analyses were performed for drain/serum bilirubin ratios on days 1, 2, 3, 4, and past day 4 including grade A to C leakages (analysis I) and consecutively for grade B and C biliary leakages (analysis II). ROC analysis was performed to determine a cutoff for the drain/serum bilirubin ratios for grade A/B/C biliary leakages after liver resections.

**Table 1:** Patient, procedure, and outcome characteristics

Patient, procedure, and outcome characteristics		Cohort (*n* = 576)	Major resection (*n* = 269)	Minor resection (*n* = 307)	*p*-value
Demographics	Age (years)	60.5 ± 13.7	61.7 ± 13.4	59.5 ± 14.0	0.06
	Gender (f/m)	249 (43.2%) 327 (56.8%)	107 (39.8%) 162 (60.2%)	142 (46.2%) 165 (53.8%)	0.12
	BMI	26.1 ± 4.9	26.4 ± 4.7	25.8 ± 5.0	0.27
Concomitant disease	ASA score	2.1 ± 0.8	2.1 ± 0.8	2.0 ± 0.8	0.24
	Chronic renal insufficiency	30 (5.2%)	15 (5.6%)	15 (4.8%)	0.71
	Cardiovascular disease	209 (36.3%)	105 (39.0%)	104 (33.8%)	0.27
	Diabetes mellitus	98 (17.0%)	56 (20.8%)	42 (13.7%)	0.02
	COPD	16 (2.8%)	7 (2.6%)	9 (2.9%)	0.81
	Viral hepatitis	76 (13.2%)	25 (0.9%)	51 (16.6%)	0.01
Liver specifics	Dignity (benign/malignant)	117 (20.3%) 459 (79.7%)	40 (14.9%) 229 (85.1%	77 (25.1%) 230 (74.9%)	0.002
	Tumor (primary/secondary)	360 (62.5%) 216 (37.5%)	168 (62.4%) 99 (37.6%)	179 (58.3%) 117 (41.7%)	0.55
	Biliary benign Echinococcus Dysontogenetic cyst IgG4 + -cholangitis Primary benign Trauma CRLM HCC iCC pCC Gallbladder-carcinoma Non-CRLM Sarcoma and others	11 (1.9%) 15 (2.6%) 28 (4.9%) 7 (1.2%) 54 (9.3%) 3 (0.5%) 147 (25.5%) 133 (23.1%) 61 (10.6%) 50 (8.7%) 6 (1.0%) 52 (9.0%) 9 (1.5%)	7 (2.6%) 8 (2.9%) 5 (1.9%) 3 (1.1%) 16 (5.9%) 0 73 (27.1%) 48 (17.8%) 43 (15.9%) 39 (14.5.%) 3 (1.1%) 20 (7.4%) 4 (1.5%)	4 (1.3%) 7 (2.2%) 23 (7.5%) 4 (1.3%) 38 (12.4%) 3 (0.9%) 74 (24.1%) 85 (27.7%) 18 (5.9%) 11 (3.5%) 3 (0.9%) 32 (10.4%) 5 (1.6%)	< 0.001
	CTx prior to resection	150 (26%)	74 (27.5%)	76 (24.8%)	0.45
	Lab values prior to resection				
	Bilirubin (mg/dL)	1.1 ± 2.1	1.1 ± 2.6	0.9 ± 1.5	0.19
	Creatinine (mg/dL)	1.1 ± 0.8	1.1 ± 0.8	1.1 ± 0.8	1.0
	INR	1.0 ± 0.1	1.0 ± 0.1	1.0 ± 0.1	0.01
	Platelets (/nL)	242 ± 95	257 ± 92	228 ± 96	< 0.001
	AST (IU/L)	49.3 ± 48.6	53.8 ± 58.2	45.0 ± 37.1	0.06
	ALT (IU/L)	50.1 ± 67.6	59.6 ± 88.4	41.8 ± 39.7	0.002
Surgical specifics	IS (min)	181 ± 94	224 ± 97	143 ± 71	< 0.001
	Major/minor	307 (53.3%) 269 (46.7%)			
	Trisectionectomy left Trisectionectomy right Hepatectomy right ext Hepatectomy left ext Hepatectomy right Hepatectomy left ≥ 1 segment ± atypical atypical Mesohepatectomy Pericystectomy + atypical Biliary duct + BDA	2 (0.3%) 33 (5.7%) 32 (5.5%) 24 (4.1%) 104 (18.8%) 62 (10.8%) 193 (33.5%) 93 (16.1%) 5 (0.8%) 17 (2.9%) 11 (1.9%)	2 (0.7%) 33 (12.3%) 32 (11.8%) 24 (8.9%) 104 (38.7%) 62 (23.0%) 6 (2.2%) 0 5 (1.4%) 1 (0.3%) 0	0 0 0 0 0 0 187 (60.9%) 93 (30.3%) 0 16 (5.2%) 11 (3.5%)	< 0.001
	BDA	75 (13%)	62 (23%)	13 (4.2.%)	< 0.001
	Pancreatic resection	5 (0.9%)	4 (1.4%)	1 (0.3%)	0.13
	Bowel resection	28 (4.9%)	10 (3.7%)	18 (5.8%)	0.23
	Pringle	200 (34.7%)	113 (42%)	87 (28.3%)	0.001
	Pringle time (min)	11.1 ± 11.5	12 ± 12	10 ± 11	0.08
Perioperative parameter	RBC within first 24 h	106 (18.4%)	61 (22.7%)	45 (14.6%)	0.01
	RBC units first 24 h	1.4 ± 4.1	1.8 ± 5.3	0.9 ± 2.0	0.06
	Complications	301 (52.3%)	175 (65%)	126 (41.0%)	< 0.001
	Dindo-Clavien ≥ 3b	109 (18.9%)	63 (23.4%)	46 (14.9%)	0.01
	Hospital stay (days)	18 ± 15	22 ± 17	15 ± 12	< 0.001
	90-day mortality	39 (6.8%)	28 (10.4%)	11 (3.6%)	0.001

**Table 2:** Bile leakage documentation

Bile leakage documentation		Cohort (*n* = 576)	Major resection (*n* = 269)	Minor resection (*n* = 307)	*p*-value
Bile leakage ISGLS (type A–C)		109 (18.9%)	65 (24.1%)	44 (14.3%)	0.003
Bile leakage ISGLS (type B/C)		96 (16.5%)	56 (20.8%)	40 (13.0%)	0.02
White test		157 (27.3%)	113 (42.0%)	44 (14.3%)	< 0.001
Intraoperative bile leakage		85 (14.8%)	63 (23.4%)	22 (7.1%)	< 0.001
Drain		442 (76.7%)	231 (85.9%)	211 (68.7%)	< 0.001
Day 1	Bilirubin S/D ratio	19.5 ± 36.9	26.5 ± 44.8	10.5 ± 23.7	
	Drain removed	9 (2.0%)	4 (1.7%)	5 (2.3%)	0.28
	Stenting/drain	3 (0.5%)	3 (1.1%)	0	0.06
	Macro. bile in drain or wounds?	12 (2.1%)	8 (2.9%)	4 (1.3%)	0.17
Day 2	Bilirubin S/D ratio	5.5 ± 13.7	6.0 ± 17.3	4.1 ± 3.5	0.41
	Drain removed	45 (7.8%)	24 (10.3%)	21 (9.9%)	0.27
	Stenting/drain	6 (1.0%)	5 (2.1%)	1 (0.3%)	0.07
	Macro. bile in drain or wounds?	20 (3.5%)	14 (5.2%)	6 (1.9%)	0.03
Day 3	Bilirubin S/D ratio	3.6 ± 8.9	3.1 ± 9.0	4.2 ± 8.9	0.07
	Drain removed	170 (29.5%)	99 (42.9%)	71 (33.6%)	0.01
	Stenting/drain	6 (1.0%)	4 (1.4%)	2 (0.6%)	0.32
	Macro. bile in drain wounds?	33 (5.7%)	23 (8.5%)	10 (3.2%)	0.006
Day 4	Bilirubin S/D ratio	3.4 ± 9.6	4.3 ± 12.1	2.3 ± 4.9	0.33
	Drain removed ≥ day4	147 (25.5%)	89 (38.5%)	58 (27.5%)	0.19
	Stenting/drain	6 (1.0%)	6 (2.2%)	0	0.04
	Macro. bile in drain or wounds?	28 (4.9%)	19 (7.0%)	9 (2.9%)	0.02
> Day 4	Bilirubin S/D ratio > day 4	4.7 ± 9.7	4.6 ± 9.9	5.0 ± 9.5	0.80
	Stenting/drain	55 (9.5%)	30 (11.1%)	25 (8.1%)	0.13
Drain longer than 7 days in situ		82 (14.2%)	51 (18.9%)	31 (10.1%)	0.002
Reoperation for bile leakage		12 (2.1%)	9 (3.3%)	3 (0.9%)	0.02

To estimate the power, sample size calculations for the validity of findings were made based on the occurrence of a bile leakage in accordance with the definition of the ISGLS for the serum bilirubin to drain ratio; we estimated that in accordance with Hanley and McNeil [12], an AUROC of 0.75, assuming the range of a 95% confidence interval of 0.18, given a power of 95%, the one-sided log test of significance with *p* = 0.05 requires the analysis of at least 338 patients [12].

Differences in demographics were detected using paired *t* tests, Fisher’s exact test, and the Pearson *Χ*^2^ test. Demographic data are given as means with standard deviation or distribution in % between the groups. Ninety-day overall survival data are given as a percentage of mortality on day 0 after the trigger surgical procedure. Univariate and multivariate analyses were performed using COX regression analysis with stepwise backward elimination. *P* values < 0.05 were defined as statistically significant. Data was analyzed with SPSS Version 25.0 (IBM, NY, USA).

Data are reported following the STROBE reporting guidelines for cohort studies [13].

## Results

### Patients

A total of 576 patients were included in this study, of which 269 (46.7%) patients had a major (> 3 segments) (group 1) and 307 (53.3%) had a minor (≤ 3 segments) (group 2) resection. All patients operated on between Jan 1, 2011, to Dec 31, 2016, were included. Demographics as well as procedural and outcome characteristics are presented in Tables 1 and 2. Bile leakage data is shown in Table 3.

**Table 3:** Univariate and multivariate analysis of binary factors predicting bile leakage types B and C

	Bile leakage cohort (*n* = 576)	Bile leakage major resection (*n* = 269)	Bile leakage cohort (*n* = 576)			Bile leakage major resection (*n* = 269)		
	Chi^2^	Chi^2^	HR	95% CI	*p*-value	HR	95% CI	*p*-value
Age > 65 years	0.04	0.01	1.51	0.9–2.4	0.07	1.7	0.9–3.2	0.09
Sex	0.76	0.52						
Chronic renal failure	0.2	0.1				0.25	0.03–2.3	0.23
Cardiovascular disease	0.15	0.39	1.1	0.7–1.8	0.64			
Diabetes mellitus	0.19	0.05	0.5	0.3–0.9	0.04	0.31	0.1–0.8	0.01
COPD	0.19	0.54	0.2	0.03–1.6	0.14			
Viral hepatitis	0.26	0.32						
Benign indication	0.97	0.88						
Primary tumor	0.04	0.02	0.8	0.5–1.4	0.14	0.91	0.4–1.9	0.82
CTx prior to surgery	0.02	0.001	0.5	0.3–0.9	0.03	0.3	0.1–0.7	0.005
BDA	< 0.001	< 0.001	2.9	1.6–5.1	< 0.001	1.88	0.9–3.7	0.07
Pringle maneuver	0.63	0.2						
RBC	0.03	0.03	1.5	0.9–2.6	0.13	1.9	0.9–3.9	0.08
White test	0.51	0.006				0.98	0.4–2.1	0.971
Intraoperative bile leakage detected	0.14	0.69	1.9	1.1–3.5	0.03			
Drain	< 0.001	0.001	5.5	2.3–13.1	< 0.001	11.8	1.5–90.6	0.02
Major resection	0.003	N.A	1.1	0.7–1.2	0.8	N.A	N.A	N.A

Univariate and multivariate analysis of binary factors predicting bile leakage types B and C (ISGLS) in the whole cohort (*n* = 576) and bile leakage types B and C (ISGLS) in patients undergoing major resection (*n* = 269). Univariate analysis was performed by chi^2^ testing. Variables with a *p* ≤ 0.09 were entered in the binomial logistic regression model. Data for multivariate regression analysis are given as hazard ratio (HR) with 95% confidence intervals (95% CI). *P*-values ≤ 0.05 were considered statistically significant. *d*, day; *COPD*, chronic obstructive pulmonary disease; *CTx*, chemotherapy; *BDA*, biliodigestive anastomosis; *RBC*, red blood cells; *HR*, hazard ratio; *CI*, confidence interval

### Demographic data

There were no significant differences between the groups regarding age, gender, body mass index (BMI), the American Society of Anesthesiologists score (ASA score), presence of renal failure, chronic obstructive pulmonary disease (COPD), or cardiovascular disease as comorbidities. In both groups, in about a quarter of cases, patients underwent a chemotherapy prior to resection depending upon the primary diagnosis (74 (27.5%) versus 76 (24.8%)). Preoperative bilirubin and creatinine levels were also comparable. In the group with a major liver resection, there was a significantly higher prevalence of diabetes mellitus (*p* = 0.02) and a significantly lower prevalence of viral hepatitis (*p* = 0.01).

### Surgical specifics and perioperative parameters

The minor resection group had a significantly lower prevalence of malignant indications (74.9% vs. 85.1%). There was no significant difference between groups regarding the status of primary or secondary tumors. Moreover, there was no deviation in the number of simultaneously performed pancreatic and bowel resections. In the major resection group, the instances in which the pringle maneuver was used (42% vs. 28.3% in the minor resection group) and in which a biliodigestive anastomosis (23% vs. 4.2% in the minor resection group) was formed were significantly higher (*p* = 0.001). The overall complication rate (according to Clavien-Dindo) was significantly higher in the major resection group (175 (65%) vs. 126 (41%), *p* < 0.001). The number of major complications (Clavien Dindo ≥ 3b) was also higher (63 (23.4%) vs. 46 (14.9%), *p* = 0.01). Additionally, the length of hospital stay was 22 ± 17 days in the major resection group and 15 ± 12 days in the minor resection group (*p* < 0.001). Subsequently, the 90-day mortality rate was higher in the major resection group (28 (10.4%) vs. 11 (3.6%), *p* = 0.001).

### Bile leakage documentation

During surgery, a bile leakage was detected either by the white test or by inspection on the surface of the liver resection plane or the hepaticojejunostomy. This was the case in 14.8% (85) of the cases and led to immediate intraoperative surgical revision (Table 2). An intraoperative bile leak occurred significantly more often in the major resection group (23.4% vs. 7.1%, *p* < 0.001). A drain was placed in 76.7% of the cases, in 85.9% of the major resection group, and in 68.7% of the minor resection group (*p* < 0.001). There were significantly more severe bile leakages (ISGLS type B/C) in the major resection group (20.8% vs. 13%, *p* = 0.02) and more bile leakages overall (ISGLS types A–C) (24.1% vs. 14.3%, *p* = 0.003).

### Management of biliary leakage

In most of the cases, the drain was removed on postoperative day 3 (29.5%) (Table 2). In total, 14.2% of the patients had their drain for more than 7 days. Significantly more of those patients had a major resection (18.9% vs. 10.1%, *p* = 0.002). Reoperation was done in 9 patients in the major resection group (3.3%) and in 3 patients in the minor resection group (0.9%) due to bile leakage type C (*p* = 0.02).

### Univariate analysis for risk factors

A univariate analysis was performed for the whole cohort (*n* = 576) and in patients undergoing major resection (*n* = 269) (Table 3). In the whole cohort risk factors for bile leakages, types B and C were age > 65 years (*p* = 0.01) and diabetes mellitus (*p* = 0.05). A primary liver tumor as diagnosis (*p* = 0.02) and prior chemotherapy to surgery (*p* = 0.001) were also significant risk factors for biliary leakage. Other factors were a BDA (*p* < 0.001), the red blood cell count (*p* = 0.03), and the placing of a drain (*p* < 0.001). In addition, statistically significant was a major resection (*p* = 0.003). Results in the major resection group showed age (*p* = 0.01), diabetes mellitus (*p* = 0.05), a primary liver tumor (*p* = 0.02), and chemotherapy (*p* = 0.001) as common risk factors. Moreover, a BDA (*p* < 0.001), the red blood cell count (*p* = 0.03), and the placing of a drain (*p* = 0.001) were shown as significant factors. In contrast to the whole group, the white test (*p* = 0.006) was another risk factor in the major resection group. Other factors mentioned in Table 3 showed no statistical significance.

### Multivariate analysis for risk factors

Variables with a *p* ≤ 0.09 were entered in the binomial logistic regression model. Multivariate analysis was performed to determine independent risk factors for the 90-day overall survival and bile leakages types B and C (ISGLS). Analysis again was performed in the whole cohort and the major resection group (Table 3). In the whole cohort, patients with diabetes mellitus (HR 0.5, 95% CI 0.3–0.9, *p* = 0.04) and prior chemotherapy (HR 0.5, 95% CI 0.3–0.9, *p* = 0.03) had a lower risk for biliary leakage. On the contrary, an intraoperative bile leakage (HR 1.9, 95% CI 1.1–3.5, *p* = 0.03), a biliodigestive anastomosis (HR 2.9, 95% CI 1.6–5.1, *p* < 0.001), and a placed drain (HR 5.5, 95% CI 2.3–13.1, *p* < 0.001) were found to be significantly associated with a higher rate of postoperative biliary leakage (Table 3). Furthermore, in the major resection group, diabetes mellitus (HR 0.31, 95% CI 0.1–0.8, *p* = 0.01) and prior chemotherapy (HR 0.3, 95% CI 0.1–0.7, *p* 0.005) were also protective factors. The placing of a drain was the only significant risk factor (HR 11.8, 95% CI 1.5–90.6, *p* = 0.02) in this group. Other factors were not statistically significant.

### ROC analysis to determine a cutoff

**Fig. 1:**
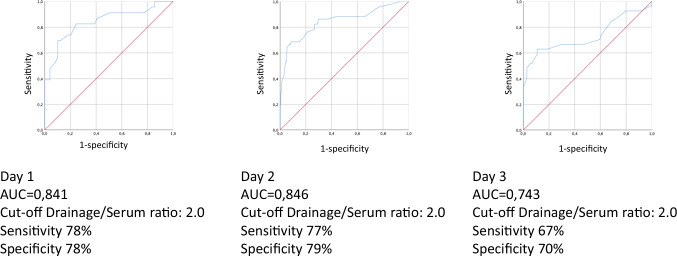
ROC analysis to determine a cutoff for the drain/serum bilirubin ration for grade A/B/C biliary leakages after liver resections

**Fig. 2:**
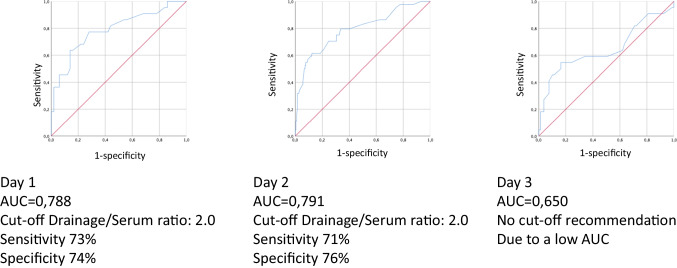
ROC analysis to determine a cutoff for the drain/serum bilirubin ration for grade B/C biliary leakages after liver resections

Areas under the curve for analysis I (grade A/B/C biliary leakage) were 0.841 (day 2), 0.846 (day 3), and 0.743 (day 4) (Fig. 1). The highest sensitivity (78% on day 1/77% on day 2) and specificity (78% on day 1/79% on day 2) were obtained for a drain/serum bilirubin ratio of 2.0 on days 1 and 2 after hepatic resection. Areas under the curve for analysis II (grade B/C biliary leakage) were similar to the ones for analysis I: 0.788 (day 1), 0.791 (day 2), and 0.650 (day 3). The highest sensitivity (73% on day 1/71% on day 2) and specificity (74% on day 1/76% on day 2) in this analysis were also detected for a drain/serum bilirubin ratio of 2.0 on postoperative days 1 and 2 (Fig. 2).

## Discussion

The current definition of bile leakage by the ISGLS which proposes a drain/serum bilirubin ratio greater than 3.0 on postoperative day 3 as a marker [14] reflects a good clinical decision tool but could be adjusted to a more precise definition given our presented results. The highest sensitivity and specificity for bile leakages were obtained for a drain/serum bilirubin ratio of 2.0 on day 2 after hepatic resection.

Biliary leakage leads to longer hospital stays and higher costs for the health care system [6]. Moreover, relevant evidence suggests that complications such as biliary leakages decrease the prognosis of patients undergoing hepatobiliary surgeries [15–17].

In the present study, bile leakage occurred in 109 (18.9%) patients, of which 96 (88%) had a severe leakage (ISGLS types B, C). This led to reoperation in 12 (11%) cases. Other studies present similar rates of bile leakages. Koch et al. [14] found biliary leakage rates of 16% in a cohort of 70 patients. Grades A, B, and C bile leakages were present in 4 (6%), 6 (9%), and 1 (1%) patients [14]. Rahbari et al. [18] included 265 patients and showed 18.4% types B and C and 27.1% types A–C bile leakages. They defined bile leakage according to the ISGLS definition by refining the findings by Koch et al. [14] Brooke-Smith et al. [19] conducted an international multicenter study with 949 included patients. Sixty-nine (7.3%) showed a biliary leakage with 31 (3.3%), 32 (3.4%), and 6 (0.6%) classified as grades A, B, and C according to the ISGLS definition. They further confirmed that a higher grade of bile leakage correlated with a higher class of postoperative complication graded by the Clavien-Dindo classification [19]. Rössler et al. [20] state a similar rate of biliary complications in 18% of 5202 hemi-hepatectomies from living donors; therefore, the shown results are very close to the benchmark. Thirty-two percent of major complications were seen to be biliary [20]. de Castro et al. [7], who used a different definition of bile leakage (bile-stained fluid (> 50 ml) in the abdominal drain more than 24 h after surgery, proven radiologically or at relaparotomy), showed rates of 2.3% [7]. Moreover, Erdogan et al. [11] who also used a different definition than the one proposed in our study (the presence of persisting bile-stained effluent from an abdominal drain, leakage detected on radiological imaging (ERC), and occurrence of a bile collection drained percutaneously or found during relaparotomy) showed a 6.8% rate (16/234) [11]. In contrast to the ISGLS definition, Nagano et al. [4] proposed a grading according to fistulogram results. They showed a 5.4% incidence rate of biliary leakage in 313 included patients [4]. They defined biliary leakage either as drainage of bile from the abdominal wound and drain, with the level of total bilirubin in the discharge fluid more than 5 mg/ml or three times the serum level or as intraabdominal collection of bile confirmed by percutaneous drainage. However, they additionally included the topography of the leakage reflected by cholangiographic evidence. They used fistulograms to directly locate the origin of the bile leakage and analyzed its prognostic value. They predict a more severe outcome for patients with peripheral bile duct involvement [4]. In comparison to the discussed results, the rates reported in this publication fall into the range with other published biliary leakage rates.

Prophylactic abdominal percutaneous drain placement after liver surgery has been seen to be possibly harmful and non-necessary in a large study group of 538 assessed by Wong-Lun-Hing et al. [21]. Drainage placement in elective liver surgery has been seen to be unnecessary due to no significant differences in mortality of complication rates [22]. Similarly, no benefits were shown in a systematic review of the efficacy of prophylactic abdominal drain after major liver resections [23]. Due to the mentioned publications, prophylactic drain placement has become less during the last years. In our cohort, 76% of patients received a drain. The ISGLS’ definition necessitates the existence of a drain and could possibly become less important in the future if drain placement was going to become less frequent.

We also aimed to investigate various factors as potential risk factors for biliary leakage. Specific surgical techniques such as biliodigestive anastomosis (BDA) are associated with higher rates of biliary leakage [24]. This could be confirmed in our analysis. The requirement for reconstructing the biliary drain with a BDA was significantly associated with a higher risk of biliary leakage in the whole cohort. Nagano et al. [4] and Erdogan et al. [11] also confirmed a higher age and a major resection as risk factors which could also be confirmed in this cohort of patients [4, 11].

Other risk factors were a drain and the detection of intraoperative bile leakage. The standard of care is to use capillary drains, not gravity drains. These findings are partly supported by Erdogan et al. [11]. Moreover, there are several studies that showed a higher occurrence of biliary leakage in patients with higher blood loss [11, 25]. Diverging results indicated operation time, number of white blood cells, and a large incisional area as specific risk factors and the usage of fibrin glue and the characteristic of liver cirrhosis as protective factors [1, 4, 5, 26]. We also identified protective factors in our multivariate analysis, such as diabetes mellitus or prior chemotherapy. How these factors can be preventing biliary leakage remains unclear and maybe accounts for patient selection and adjusting the operative procedures to the underlying concomitant factors. Differences in rates of biliary leakage or in rates of mortality may be due to multiple possible biasing factors such as the sample size, the primary indications or resections, and the used definition of bile leakage. Further research regarding the outcomes of patients with bilirubin serum to drain ratios that are already higher on day 2 may be conducted, but were not the purpose of this study.

Limitations of the current study are its retrospective nature, although an unselected cohort of consecutively operated patients was included. Using the same computed patient records in our hospital, the error in collecting data should be marginal. The proposal was based on objective patient data to ensure external validity. The study population consisted of all patients who underwent a liver resection during the years 2011 to 2016 at the University Hospital Frankfurt. Consequently, selection bias should be negligible. Laboratory values before resection differ from day to day and therefore only portray the current state and could therefore lead to biased results.

Clinically relevant bile leakages were treated with ERCP or surgically. There exist retrospective analyses of ERCP in patients with bile leakage stating no difference in time point (one day or several days). Emergency ERCP on the same day had higher complication rates [27, 28]. We did not analyze this aspect in this cohort. Bile leakage grade A according to the definition requires no or little change in the patient’s clinical management but can become an important factor in a patient’s treatment. Nevertheless, there may be a consideration to change definitions of this type such as the definition of the postoperative pancreatic fistula grade A, which has been changed to biochemical leak after the 2016 revision [29].

## Conclusion

The findings regarding our single-center study do not contradict the results of the ISGLS, but regarding all our findings, we show significantly better probabilities for a biliary leakage with the highest sensitivity and specificity when extracting the bilirubin level in the drain at postoperative day 2 compared to day 3. That means that 24 h prior to our momentarily used approach, we can already detect biliary leakage and initiate a different clinical approach if necessary. This has a potential for earlier treatment and could therefore reduce further complications, the length of stay, and hospital costs.

The ISGLS definition of biliary leakage could be confirmed in the current study, and the knowledge that already a factor 2 on day 2 implies biliary leakage as diagnosis may lead to a clinically relevant change of therapy and therefore represents an important refinement of the ISGLS definition, also giving space for sooner action to resolve the leakage. However, the location and topography of biliary leakages such as the Nagano classification should be included in the ISGLS definition in the future to better estimate the therapeutic need to resolve the leakage or if a spontaneous healing can be suspected.

## Data Availability

The anonymized data set confirming the outcomes of the study can be requested from the authors.

## Abbreviations

ASA: American Society of Anesthesiologists
BDA: Biliodigestive anastomosis
BMI: Body mass index
CI: Confidence interval
CLRM: Colorectal liver metastasis
COPD: Chronic obstructive pulmonary disease
CTx: Chemotherapy
D: Day
ERCP: Endoscopic retrograde cholangiopancreatography
ERC: Endoscopic retrograde cholangiography
HCC: Hepatocellular carcinoma
HR: Hazard ratio
iCC: Intrahepatic cholangiocellular carcinoma
IS: Incision suture time
ISGLS: International Study Group of Liver Surgery
OS: Overall survival
pCCC: Perihilar cholangiocellular carcinoma
RBC: Red blood cells
S/D: Serum/drain ratio
